# Body Mass Index and Waist Circumference as Predictors of Above-Average Increased Cardiovascular Risk Assessed by the SCORE2 and SCORE2-OP Calculators and the Proposition of New Optimal Cut-Off Values: Cross-Sectional Single-Center Study

**DOI:** 10.3390/jcm13071931

**Published:** 2024-03-27

**Authors:** Szymon Suwała, Roman Junik

**Affiliations:** Department of Endocrinology and Diabetology, Nicolaus Copernicus University, Collegium Medicum, 9 Skłodowskiej-Curie Street, 85-094 Bydgoszcz, Poland; junik@cm.umk.pl

**Keywords:** obesity, abdominal obesity, cardiovascular risk, SCORE2

## Abstract

**Background:** Obesity has been perceived as one of the important cardiovascular risk factors, but SCORE2 calculators used in clinical practice do not include the most popular parameters assessed for body composition: body mass index (BMI) and waist circumference (WC). The objective of this research was to determine which of the aforementioned variables is a more reliable predictor of an above-average increased cardiovascular risk for gender and age (ICVR). **Methods:** Data from 2061 patients were analyzed; the 10-year risk of cardiovascular events was assessed by SCORE2 tables, and the correlations with BMI and WC were analyzed. **Results:** BMI and WC independently predicted ICVR (OR 1.10–1.27). In males, BMI was a more accurate predictor (AUC = 0.816); however, in females, it was WC (AUC = 0.739). A novel threshold for BMI (27.6 kg/m^2^) was suggested, which increases the risk of cardiovascular disease by 3.3–5.3 times depending on gender; the same holds true for WC (93 cm in women and 99 cm in men; 3.8–4.8-fold higher risk). **Conclusions:** Despite their heterogeneity, BMI and WC are effective cardiovascular risk predictors, especially BMI for males and WC for females; therefore, more research is needed to include them in future models for predicting unfavorable cardiometabolic events.

## 1. Introduction

Obesity is a prevalent issue on a global scale, strongly associated with the occurrence of noncommunicable diseases such as hypertension, cardiovascular disorders, and diabetes. Based on a report by the World Health Organization, a commonly used definition of obesity is determined by body mass index (BMI), calculated by dividing weight in kilograms by height in meters squared. In the Caucasian population, a BMI of 25 kg/m^2^ or higher is generally considered overweight, while a BMI of 30 kg/m^2^ or higher indicates obesity [1,2]. However, it was commonly observed that BMI is not a perfect measure. The significance of recognizing abdominal obesity through waist circumference (WC) was highlighted, as it is linked to the development of cardiometabolic and cardiovascular diseases in individuals with “normal-weight obesity”, which refers to individuals who have excess fat but do not meet the criteria for obesity based on their BMI [3]. This was evident in the series of criteria published for the metabolic syndrome, which recognized the important role of obesity in the development of cardiovascular diseases. However, these criteria only considered the waist circumference (WC) parameter. In contrast, the latest criteria for metabolic syndrome from Polish scientific societies in 2022 have reintroduced the use of BMI as an obesitological parameter, in addition to WC (as outlined by the National Cholesterol Education Program: Adult Treatment Panel III—NCEP: ATP III) [4,5,6,7].

The aforementioned metabolic syndrome is characterized as a set of interconnected elements that increase the patient’s susceptibility to cardiovascular diseases. These factors include obesity, hypertension, and disorders in lipid and glucose metabolism, among others. Nevertheless, there is a useful tool available for predicting cardiovascular risk (specifically, 10-year fatal and non-fatal risk in individuals without previous cardiovascular disease or diabetes) known as SCORE calculators, especially after re-evaluation (SCORE2) and supplementing an assessment in older patients (SCORE2-OP), allowing for the inclusion of individuals aged 40 to 89 in the group of patients being examined [8,9,10]. These indicators are quite simple and effective, taking into account the patient’s gender, age, smoking status, blood pressure, and non-HDL cholesterol concentration—and although, for example, the criteria for the metabolic syndrome in Poland have become excessively focused on obesity, the SCORE2 and SCORE2-OP calculators do not take into account obesity and parameters assessing it in general [7].

Due to this possible contrast, we performed a study to find the better indicator (BMI or WC, which are both commonly used to measure obesity, especially according to metabolic syndrome criteria) of above-average increased cardiovascular event risk for gender and age (ICVR) and to suggest new best cut-offs for both of these metrics. ICVR represents the risk that is situated in the higher quartiles for a specific gender, age, and cigarette dependency and, therefore, falls within the SCORE2 and SCORE2-OP tables in separate, big squares. For the purpose of this study, we devised this idea to ensure that the results are not influenced by the most significant factor affecting cardiovascular risk, which is age [11]. This manuscript presents the findings of our research.

## 2. Materials and Methods

This was a single-center cross-sectional study and involved analyzing the medical records of all patients between the ages of 40 and 89 who were hospitalized at the authors’ department from 2015 to 2020 and in whom it was possible to determine the 10-year cardiovascular risk using SCORE2 and SCORE2-OP calculators (necessary data include gender, age, nicotine addiction, systolic blood pressure, and non-HDL cholesterol concentration [9,10]), who also had both BMI and WC assessed during their stay in the hospital. Following hospital regulations, the patient’s body weight was measured with a digital scale while wearing only underwear, and height was measured with a tape measure, approximating each measurement to the nearest centimeter—WC was measured using a tape on the patient’s skin, namely as the smallest circumference between the lower costal margin and the higher margin of the iliac crest; blood pressure was promptly assessed by the nursing staff subsequent to the WC measurement, and the result was immediately documented in the patient’s electronic medical records. Upon hospital admission, both nurses and physicians diligently gathered information about the patient’s current nicotine addiction to ensure accuracy and avoid any mistakes. During the analyzed period, the laboratory lipid parameters were primarily evaluated using the ARCHITECT ci8200 analyzer (Abbott Diagnostics). We excluded individuals with atherosclerotic cardiovascular disease, diabetes (SCORE2 and SCORE2-OP are not validated and calibrated for patients with diabetes, unlike SCORE2-Diabetes, which was not used in this study [12]), chronic renal disease, familial hypercholesterolemia, and any other genetic abnormalities that could potentially affect lipid metabolism or blood pressure. Considering the aforementioned rules, following an investigation of the medical records of 6185 hospitalized unique patients, data from 2061 patients (33.32%) were chosen for this study—957 men (46.43%) and 1104 women (53.57%). We received approval for our study from The Bioethics Committee of the Nicolaus Copernicus University, functioning at Collegium Medicum in Bydgoszcz (approval number 129/2019). The study was conducted following the guidelines of the Declaration of Helsinki.

To conduct the necessary analyses, we utilized the conventional criteria for overweight and obesity based on BMI for the Caucasian population (≥25 kg/m^2^ and 30 kg/m^2^, respectively). Additionally, we employed two different definitions of abdominal obesity based on WC, which were derived from the criteria used by the International Diabetes Foundation (IDF; ≥80 cm for females, ≥94 cm for males) and the NCEP: ATP III (≥88 cm for females, ≥102 cm for males) [4,5].

Regarding statistical analysis, the software Statistica 13.3, developed by StatSoft Inc. in Tulsa, OK, USA, was utilized. Continuous variables are represented by their median and interquartile range (IQR: 25–75th percentile), whereas categorical variables are reported as numbers and percentages. The Kolmogorov–Smirnov test was utilized to assess the normality of the results. The results were compared using the Fisher exact test or Chi-squared test for categorical variables and the Mann–Whitney U-test for continuous data (due to an abnormal distribution of variables). The study also examined receiver operating characteristic (ROC) curves; the area under the curve was determined and calculated with a 95% confidence interval (AUC, 95% CI), and thresholds were assessed for sensitivity (Se), specificity (Sp), negative predictive value (NPV), positive predictive value (PPV), diagnostic accuracy (DA) and Youden index (YI). YI, which is calculated as sensitivity + specificity −1 for each observed value of the predictor, was assessed by the authors as a crucial parameter in determining the most effective cut-off values—referring to general statistical principles, the largest value of YI enables the selection of the most effective cut-points, particularly when considering that YI maximizes the sum of specificity and sensitivity and reflects the probability of a positive result among patients with the condition as opposed to those without it [13,14]. The cutoff value for statistical significance was established at <0.05.

The anticipated 10-year risk of cardiovascular events and mortality for each patient was evaluated using the SCORE2 and SCORE2-OP tables for high-risk countries (like Poland) [9,10]. The median of the predicted risk was obtained for each group (shown by big squares in the tables SCORE2 and SCORE2-OP) and varies according to gender, active nicotine use, and age of patients. Patients who met the criterion for ICVR were identified as patients whose calculated risk was above the median for each category—as a result, the age and gender of patients, which have been considered the primary risk factors for cardiovascular events [11], do not affect our comprehensive evaluation of the impact of BMI or WC on cardiovascular event risk. Table 1 shows the cut-off values defined for ICVR, as mentioned before.

## 3. Results

The final cohort of participants comprised a total of 2061 individuals, with 957 being male (46.43%) and 1104 being female (53.57%). The patients had an average age of 64.12 years (IQR 54.86–73.02)—there was a significant difference in genders, with men having an average age of 62.71 years (IQR 53.33–71.80) and women 64.90 years (IQR 56.14–73.76) (*p* < 0.001). A total of 562 patients (27.27%) were smokers—the prevalence of smoking was higher among men (30.20%; *n* = 289) compared to women (24.73%; *n* = 273); the difference was found to be statistically marginally significant (*p* = 0.057). Systolic blood pressure did not differ significantly between men and women—the median was 132 mmHg (IQR: 126–139). The median non-HDL fraction cholesterol concentration was 3.3 mmol/L (IQR 2.5–4.3) and showed a significant difference between genders (*p* < 0.001): in males it was 3.1 mmol/L (IQR 2.4–4.1), while in females it was 3.4 mmol/L (IQR 2.6–4.4). The median BMI was found to be 27.82 kg/m^2^ with an IQR of 23.74–30.35, and no statistically significant difference was observed depending on gender (*p* = 0.453). Men had an average waist circumference of 99.00 cm (IQR 91.00–106.00), whereas women had an average waist circumference of 93 cm (IQR 84.0–98.0); *p* < 0.001.

Out of the total number of patients, 624 (30.28%) met the ICVR criterion—283 (29.57%) men and 341 (30.89%) women; there was no statistically significant difference observed in the age of these patients when compared to those who did not meet the ICVR requirement (*p* = 0.478). Similarly, there was no significant difference in the percentage of individuals with nicotine addiction (*p* = 0.461). However, there were noticeable differences in terms of systolic blood pressure (for males: 142 mmHg with IQR 136–149 vs. 128 mmHg with IQR 122–135, *p* < 0.001; for females: 142 mmHg with IQR 135–146 vs. 130 mmHg with IQR 124–134, *p* < 0.001) and non-HDL cholesterol concentration (for males: 4.6 mmol/L with IQR 4.0–5.4 vs. 2.7 mmol/L with IQR 2.2–3.3, *p* < 0.001; for females: 4.3 mmol/L with IQR 3.5–5.3 vs. 3.1 mmol/L with IQR 2.5–3.9, *p* < 0.001). This confirms the hypothesis that ICVR is age- and gender-independent.

Patients who met the ICVR criterion had a higher BMI and WC compared to those who did not, both in the male group (BMI: 31.36 kg/m^2^ with IQR 28.31–35.09 vs. 26.91 kg/m^2^ with IQR 23.07–28.80, *p* < 0.001; WC: 107 cm with IQR 100–116 vs. 97 cm with IQR 89–103, *p* < 0.001) and the female group (BMI: 29.90 kg/m^2^ with IQR 27.89–33.18 vs. 27.05 kg/m^2^ with IQR 23.29–28.96, *p* < 0.001; WC: 98 cm with IQR 93–105 vs. 91 cm with IQR 83–95 cm, *p* < 0.001). Both BMI and WC were found to be significant and independent factors in the multivariate logistic regression analysis, influencing the patient’s belonging to the ICVR group (for BMI: OR 1.27, 95% CI: 1.24–1.31, *p* < 0.001; for WC: OR 1.10, 95% CI: 1.09–1.11, *p* < 0.001).

**Figure 1:**
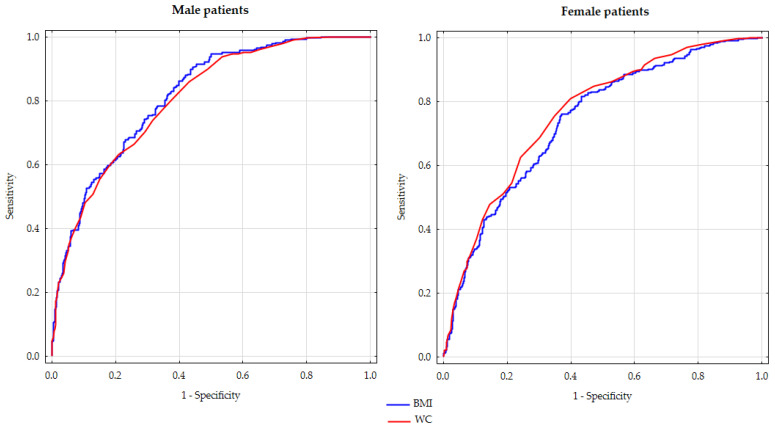
ROC curves for body mass index and waist circumference as predictors of above-average increased cardiovascular risk for gender and age in male and female patients.

The predictive abilities of BMI and WC for ICVR were assessed using ROC curves, as depicted in Figure 1. The study revealed that in males, BMI demonstrated a higher predictive capability in comparison to WC (AUC 0.816, 95%CI: 0.788–0.843 vs. AUC 0.804, 95%CI 0.775–0.833, *p* < 0.001). In contrast, in women, WC exhibited a slightly higher predictive power (AUC for BMI 0.739, 95% CI 0.708–0.770 compared to AUC for WC 0.762, 95%CI 0.732–0.791, *p* < 0.001). Table 2 provides detailed information on Se, Sp, NPV, PPV, DA, and YI for the thresholds used to classify individuals as overweight or obese based on BMI (25 kg/m^2^ and 30 kg/m^2^, respectively) and abdominal obesity according to the IDF criteria (WC 80 cm for women, 94 cm for men) and NCEP: ATPIII criteria (WC 88 cm for women, 102 cm for men)—the table also shows the same data for the most optimal cut-offs for BMI (27.6 kg/m^2^ for both genders, with YI being the highest) and WC (93 cm for women, 99 cm for men).

**Figure 2:**
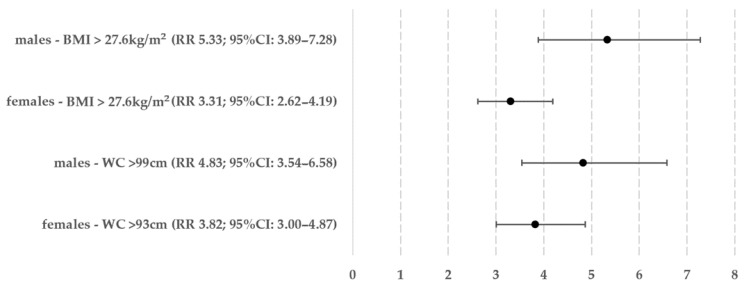
Relative above-average cardiovascular risk in women and men meeting the criteria of increased body mass index or waist circumference.

The ROC curve analysis results revealed that the optimal cut-off values for BMI and WC are 27.6 kg/m^2^ for both sexes and 93 cm for women and 99 cm for men, respectively. The frequency of meeting the ICVR criterion was then assessed in patients with values exceeding these cut-offs. Out of the women, 595 (53.89%) had an increased BMI, and among them, 271 also met the ICVR criterion (45.55% of women with an increased BMI, 24.55% of all women). In terms of WC, 581 women (52.63%) had a value greater than 93 cm, and out of them, 276 also met the ICVR criterion (47.50% of women with high WC, 25.00% of all women). Regarding men, 517 individuals (54.02%) fulfilled the requirement of elevated BMI, out of which 244 also fulfilled the ICVR requirement (47.20% of the group with high BMI, 25.50% of all men). In terms of WC, 533 individuals (55.69%) had a measurement exceeding 99 cm, of which 243 individuals simultaneously fulfilled the ICVR requirement (45.59% of men with increased WC, 25.39% of all men). The Chi-squared test results showed that there were no significant differences between genders in terms of elevated BMI and WC requirements (*p* = 0.953 for BMI, *p* = 0.163 for WC). The relative risk was estimated by comparing groups of patients who did not match the criteria for increasing BMI and WC—this information is shown in Figure 2.

## 4. Discussion

This study aimed to determine whether BMI or WC, two commonly used body measurements in the field of clinical practice and research on patients with obesity, could provide a more precise prediction of an above-average increased cardiovascular risk for gender and age. We utilized the SCORE2 and SCORE2-OP calculators for this purpose. Our research suggests that BMI and WC have different predictive qualities depending on gender—WC is a more reliable indicator for women, while BMI is more accurate for men. The difference in the risk between genders for these indicators (at their most optimal values, specifically BMI 27.6 kg/m^2^, WC 93 cm and 99 cm) was approximately 0.5 in absolute terms and 9.4–13.4% in terms of percentage (5.33 vs. 4.83 in men and 3.82 vs. 3.31 in women).

In a study similar to our own, the author aimed to determine the most effective cut-off values for BMI, WC, WHR (waist-to-hip ratio), and WHtR (waist-to-height ratio) in identifying individuals with a high body fat percentage and an increased risk of cardiometabolic disorders. The study also found separate optimal values for BMI and WC in both men and women (BMI: 28.1 kg/m^2^ for men and similar to our 27.5 kg/m^2^ for women; WC: similar to our 100 cm for men and 87 cm for women). In both genders, BMI was found to have a slightly higher value compared to WC; WHR and WHtR, which were not examined in our study, were found to have significantly lower quality values [15]. In another study, researchers from Brazil examined the relationship between body fat percentage, fat mass index, and BMI in relation to predicting different cardiometabolic risk factors such as hypertension, elevated C-reactive protein levels, hyperglycemia, or dyslipidemia—the researchers discovered that specific BMI cut-off values were effective in identifying the presence of three or more of these risk factors (for men, the optimal BMI cut-off values ranged from 27.0 to 28.4 kg/m^2^, while for women, the range was 26.9–28.5 kg/m^2^, varying by region), and the diagnostic results obtained from these cut-off values were deemed satisfactory [16]. In their study, Głuszek et al. determined that a BMI of 27.2 kg/m^2^ is the threshold for diagnosing metabolic disorders, applicable to both males and females [17]. There are other confirmations that (at least in the context of BMI) higher cardiometabolic and cardiovascular risk should be considered in cases of overweight rather than obesity, regardless of gender and age. Reaching a definitive conclusion in the case of WC is quite challenging, mainly due to the absence of a universally accepted definition for overweight and the presence of two different definitions of abdominal obesity in the European population (based on criteria set by the IDF: ≥80 cm for women, ≥94 cm for men, and the NCEP: ATP III: ≥88 cm for women, ≥102 cm for men); however, most endocrinologists and obesity specialists acknowledge the IDF criteria as superior [4,5]. In our study, the most accurate predictive value for women was 13 cm above the IDF criteria (closer to the NCEP: ATP III criteria because it differs by −1 cm), and for men, it was 5 cm above (also more similar to the criteria of NCEP: ATP III, differing by −3 cm). A study conducted by Cardinal et al. found that the most effective waist circumference cut-off for screening metabolic syndrome in women was 86 cm, while for men, it was 92 cm [18], and the Petermann-Rocha study from Chile reported comparable results of 87.6 cm for women and 92.3 cm for men [19]. We are intrigued by a unique finding by Prasad et al., who established very low thresholds for WC related to any of the cardiovascular risk factors: 77.5 cm for women and 84.5 cm for men [20]. A study conducted by Chinese researchers examined various anthropometric parameters (BMI, WC, WHR, Body Shape Index: ABSI, Abdominal Volume Index: AVI and others) to determine their ability to predict the risk of metabolic syndrome—BMI was the most effective predictor for both genders; however, due to the simplicity and widespread use of WC, which had an almost similar diagnostic accuracy to BMI, the researchers suggest using WC as the primary parameter [21]. When examining these reports collectively, it is crucial to take into account the different interpretations of cardiometabolic risk and the diverse objectives of each study. In our study, we employed our own assessment using the SCORE2 and SCORE2-OP tables, as well as an age-and-gender-independent ICVR, when other studies concentrated on specific elements of the metabolic syndrome. Regardless, it is clear that there are population disparities in the perception and discriminatory power of both BMI and WC.

Given the differences in diagnostic accuracy of BMI and WC between genders in our study, it is important to identify common characteristics that link these two measurements. In 2018, Park et al. introduced a new anthropometric measure for obesity called the weight-adjusted-waist index (WWI)—this index standardizes WC in relation to body weight and has been shown to have several advantages over traditional measures like WC and BMI (WWI is a comprehensive indicator of obesity, as it has a positive relationship with cardiometabolic disease and mortality) [22]. WWI was also associated with a higher prevalence of gallstones, non-alcoholic fatty liver disease, and liver fibrosis; these conditions are considered independent cardiovascular risk factors, particularly in individuals with comorbid obesity and diabetes (also known as diabesity) and overall multimorbidity [23,24,25,26,27]. However, studies investigating the association between WWI and cardiovascular risk are limited, and WWI has not been acknowledged in routine clinical practice—thus, it has not been considered in our current work. Moreover, it is worth contemplating the possibility of exploring the role of WWI in the Polish population of patients with overweight or obesity in the future. If such an investigation proves successful, it may be worth considering the integration of WWI into everyday clinical practice.

It is worth mentioning that no matter which predictor we used, our study found that women have a lower estimated cardiovascular risk based on age, according to the SCORE2 and SCORE2-OP calculators. Previous studies indicate that variations in body composition and the structure of fat deposits can play a role in the observed differences in outcomes. As an example, a study by Schorr et al. found that there are gender differences in cardiometabolic risk profiles based on the amount of visceral adipose tissue and the mass of lipids in certain cells in the body (the study also mentioned that adipose tissue in the lower extremities can have a protective effect). The authors concluded by noting the limitations of commonly used body measurements and suggesting using more detailed imaging techniques instead [28]. Cardiologists from the Mayo Clinic have found that the distribution of fat in the body can greatly affect the risk of heart disease and mortality in individuals with a normal body weight (those with visceral obesity had a 2.75 times higher risk of heart disease and a 2.08 times higher risk of death from any cause compared to those without visceral obesity) [29].

Our concept of age-and-gender-independent ICVR based on the SCORE2 and SCORE2-OP tables was created to verify the role of one of the two factors not included in the mentioned tables while at the same time being inherent components of the diagnosis of the metabolic syndrome: obesity and, therefore, anthropometric parameters such as BMI and WC (the second factor not taken into account in the SCORE2 and SCORE2-OP tables are prediabetes states, linked with fasting blood glucose, oral glucose tolerance test, or HbA1c percentage). The models SCORE2 and SCORE2-OP are commonly used for predicting an elevated cardiovascular risk over a 10-year period [9,10]. A study conducted by van Trier et al. validated both models and confirmed that in a population cohort from the UK, SCORE2 showed reasonable accuracy and consistency in predicting risk; however, SCORE2-OP performed poorly in discriminating and underestimated the risk for both males and females, particularly in individuals aged 70–80 years [30,31]. Nevertheless, it is futile to search for a perfect concept—according to Kasim et al., many similar predictive models have shown a tendency to overestimate the 10-year cardiovascular risk by a significant amount, ranging from 3% to 1430% [32]. In routine clinical practice, it is important for cardiovascular risk prediction models to be user-friendly (especially when considering that cardiovascular diseases are the primary contributors to mortality associated with diseases in the Polish population). Both SCORE2 and SCORE2-OP meet this requirement, which explains their popularity and our decision to use them.

Recognizing and addressing limitations is an essential aspect of any academic research, and this study is no different. The study involves a significant number of patients, but it is a very heterogeneous group—therefore, in order to verify the increased cardiovascular risk, we opted to categorize them into smaller groups based on age, gender, and nicotine addiction in accordance with the SCORE2 and SCORE2-OP tables. It is important to mention that we did not collect data on variables like insulin resistance (such as HOMA-IR), which could potentially impact the final findings (waist circumference is sometimes considered an indirect indicator of insulin resistance [33]). A significant drawback of this study is also the lack of additional anthropometric indicators, such as ABSI, Body Roundness Index, Conicity Index, WWI, BMI_WC_ or others (which are known to be beneficial in assessing cardiovascular risk [22,34,35,36])—nevertheless, this was done on purpose; the mentioned indicators are not typically used in regular clinical practice with patients who have overweight or obesity. Another concern may arise from the study’s design, which may not give the impression that obesity-related factors have an impact on blood pressure and lipid parameters. As a result, our conclusions may be considered more mathematical in nature than clinically significant. It is evident that obesity plays a central role in all aspects of the metabolic syndrome, including dyslipidemia and arterial hypertension—this has been supported by previous research [7]. In our study, we focused on assessing the impact of obesity (specifically, parameters such as BMI and WC) on dichotomously understood cardiovascular risk (higher or lower than the median for a particular subgroup of patients), where risk, in fact, using the SCORE2 and SCORE2-OP tables, is an effect of a unified and integrated function of all these variables (except for obesity)—the SCORE2 calculation principles involve age, gender, smoking status, blood pressure, and lipid assessment [9,10]; by dividing subjects into smaller cohorts (as illustrated in Table 1), BMI and WC can be utilized as surrogates for lipids and blood pressure. Of course, it would be reasonable to undertake a prospective assessment of specific cardiovascular events in the future and not only the risk assessed in a model; such a study is being considered. Our future plans involve also forming partnerships with other specialized institutes that focus on studying the consequences associated with overweight and obesity, such as cardiac, hepatological, nephrological, and others. Through a multicenter study, we aim to broaden our research in these specific areas. This study serves as a valuable beginning for future scientific discussions regarding the determinants of obesity-related cardiovascular risk and may serve as a source of inspiration for other research teams.

## 5. Conclusions

Given our innovative age-and-gender-independent ICVR concept, it is worth noting that both BMI and WC show similar diagnostic effectiveness. WC is particularly beneficial for women, while BMI slightly outperforms for men. Given this gender-dependent variation, further research is required to develop new or reconsider old parameters that integrate both BMI and WC for widespread application in clinical practice. In terms of BMI, the ICVR threshold of 27.6 kg/m^2^ applies to both genders, which indicates that we should consider cardiometabolic risk not just in cases of obesity but also in cases of overweight among patients. The WC cut-off points (93 cm for women, 99 cm for men, close to the criteria for diagnosis of abdominal obesity as per NCEP: ATP III) should be further investigated to confirm their validity; however, these cut-off points showed a significantly increased risk (3.8–4.8 times higher) of cardiovascular events and mortality over the next ten years. Considering the limited scope of our research, progressing toward a multicenter study is recommended.

## Figures and Tables

**Table 1:** Cut-offs for above-average increased cardiovascular risk for gender and age (ICVR) for every specific group based on SCORE2 and SCORE2-OP tables.

Group	Cut-Off to Define ICVR in Specific Group
Females, 40–44 years old, non-smokers	>1%
Females, 40–44 years old, smokers	>4%
Males, 40–44 years old, non-smokers	>2%
Males, 40–44 years old, smokers	>5%
Females, 45–49 years old, non-smokers	>2%
Females, 45–49 years old, smokers	>4%
Males, 45–49 years old, non-smokers	>3%
Males, 45–49 years old, smokers	>6%
Females, 50–54 years old, non-smokers	>3%
Females, 50–54 years old, smokers	>9%
Males, 50–54 years old, non-smokers	>4%
Males, 50–54 years old, smokers	>10%
Females, 55–59 years old, non-smokers	>9%
Females, 55–59 years old, smokers	>10%
Males, 55–59 years old, non-smokers	>11%
Males, 55–59 years old, smokers	>12%
Females, 60–64 years old, non-smokers	>7%
Females, 60–64 years old, smokers	>13%
Males, 60–64 years old, non-smokers	>8%
Males, 60–64 years old, smokers	>13%
Females, 65–44 years old, non-smokers	>10%
Females, 65–69 years old, smokers	>16%
Males, 65–69 years old, non-smokers	>11%
Males, 65–69 years old, smokers	>17%
Females, 70–74 years old, non-smokers	>14%
Females, 70–74 years old, smokers	>27%
Males, 70–74 years old, non-smokers	>15%
Males, 70–74 years old, smokers	>29%
Females, 75–79 years old, non-smokers	>22%
Females, 75–79 years old, smokers	>28%
Males, 75–79 years old, non-smokers	>23%
Males, 75–79 years old, smokers	>33%
Females, 80–84 years old, non-smokers	>31%
Females, 80–84 years old, smokers	>40%
Males, 80–84 years old, non-smokers	>29%
Males, 80–84 years old, smokers	>42%
Females, 85–89 years old, non-smokers	>47%
Females, 85–89 years old, smokers	>52%
Males, 85–89 years old, non-smokers	>43%
Males, 80–89 years old, smokers	>50%

**Table 2:** Analysis of ROC curves for body mass index and waist circumference as predictors of above-average cardiovascular risk for gender and age, with the most optimal cut-offs and thresholds traditionally adapted for the diagnosis of overweight, obesity, and abdominal obesity.

Cut-Off Point	Group (Males/Female)	Se [%]	Sp [%]	NPV [%]	PPV [%]	DA [%]	YI
Body mass index							
25.0 kg/m^2^	Males	95.8	60.2	95.7	40.0	56.3	0.355
	Females	89.7	38.5	89.4	39.5	54.3	0.283
27.6 kg/m^2^	Males	86.2	60.1	91.2	47.6	67.8	0.463
	Females	75.4	36.6	85.2	47.9	67.1	0.388
30.0 kg/m^2^	Males	58.0	83.5	82.6	59.6	76.0	0.415
	Females	47.8	82.3	77.9	54.7	71.6	0.301
Waist circumference							
80 cm	Females	99.7	7.5	98.3	32.5	36.0	0.072
88 cm	Females	89.7	39.1	89.5	39.7	54.7	0.288
93 cm	Females	80.9	60.0	87.6	47.5	66.5	0.410
94 cm	Males	95.1	39.8	95.0	39.9	56.1	0.348
99 cm	Males	85.9	57.0	90.6	45.6	65.5	0.428
102 cm	Males	70.3	70.8	85.6	50.3	70.6	0.411

## Data Availability

The data can be made available upon reasonable request—please contact the correspondence author. The data are not publicly available because they contain information that could compromise the privacy of research participants.
